# Health Promotion in an Age of Normative Equity and Rampant Inequality

**DOI:** 10.15171/ijhpm.2016.95

**Published:** 2016-07-18

**Authors:** Ronald Labonté

**Affiliations:** Canada Research Chair, Globalization and Health Equity, Faculty of Medicine, School of Epidemiology, Public Health and Preventive Medicine, University of Ottawa, Ottawa, ON, Canada.

**Keywords:** Health Promotion, Sustainable Development Goals (SDGs), Inequalities, Climate Change, Neoliberalism

## Abstract

The world was different when the Ottawa Charter for Health Promotion was released 30 years ago. Concerns over the environment and what we now call the ‘social determinants of health’ were prominent in 1986. But the acceleration of ecological crises and economic inequalities since then, in a more complex and multi-polar world, pose dramatically new challenges for those committed to the original vision of the Charter. Can the 2015 Sustainable Development Goals (SDGs), agreed to by all the world’s governments, offer a new advocacy and programmatic platform for a renewal of health promotion’s founding ethos? Critiqued from both the right and the left for, respectively, their aspirational idealism and lack of political analysis, the SDGs are an imperfect but still compelling normative statement of how much of the world thinks the world should look like. Many of the goals and targets provide signals for what we need to achieve, even if there remains a critical lacuna in articulating how this is to be done. The fundamental flaw in the SDGs is the implicit assumption that the same economic system, and its still-present neoliberal governing rules, that have created or accelerated our present era of rampaging inequality and environmental peril can somehow be harnessed to engineer the reverse. This flaw is not irrevocable, however, if health promoters – practitioners, researchers, advocates – focus their efforts on a few key SDGs that, with some additional critique, form a basic blueprint for a system of national and global regulation of capitalism (or even its transformation) that is desperately needed for social and ecological survival into the 22nd century. Whether or not these efforts succeed is a future unknown; but that the efforts are made is a present urgency.

It has been 30 years since the *Ottawa Charter for Health Promotion*^1^ became a ‘new public health’ manifesto that echoed around the world. Emanating first from high-income countries (HICs), its simple messaging (‘enable, mediate, advocate’) and emphasis on ‘basic prerequisites for health’ – today’s ‘social (or political, or commercial) determinants of health’ – quickly diffused globally. A few years after its release I suggested that its rhetorical rise lay partly in how it embodied the aspirations of progressive social movement activists from the 1970s who had moved into positions of public health authority in the 1980s, pushing against the confines of bureaucratic conservatism.^2^ More nuanced explanations invoking public policy theories could also be advanced; but it was fundamentally a heady, optimistic time for a politically progressive push around a health promotion agenda that transcended the educationalist focus on ‘lifestyles.’^3^

It was a different world then from now. Geopolitical power remained divided between the capitalist and communist world. The Berlin Wall still stood. Fundamentalist Islam had yet to enter the vacuum created by the collapse of the Soviet Union. The austerity of neoliberal economics had just started its global ascension in the structural adjustment programs that bailed out international banks caught in the 1980s’ developing world debt crisis.^4^ BRICS (Brazil, Russia, India, China, and South Africa countries) referred to building material, outsourcing had yet to become a standard dictionary entry, financialization of the global economy had barely started, extreme weather referred to an occasional storm, and 1% was simply an uninteresting percentile. It is trite, but not inaccurate, to say that the past three decades have witnessed tectonic shifts in the global political and economic determinants of health. The contemporary world finds itself poised between two great antinomies. On the one hand we have a rampant inequality in which the ever-shrinking 0.000 000 01% of humanity – just 62 people – now hold as much global wealth and the resources and political power it commands as the rest of the planet’s 7.4 billion residents,^5^ and whose ecological footprint is 175 greater than the world’s ‘bottom billion.’^6^ On the other is the parallel rise in global normative equity, the ever more public and governmental commitments to greater fairness in the distribution of resources essential for life now, and for future generations.

How are those whose vocation is to improve equitable population health (‘levelling up’ the bottom) to negotiate such dissonance? What should a health promotion ethos and practice look like in an era of anthropogenic depredation, economic stagnation and a ‘liquid modernity’^7^ that challenges the possibility of collective politics, all too easily devolving all responsibility to the individual?

## A Pre-apocalyptic 2015

It would be naïve to assume the zesty hopefulness of the *Ottawa Charter*, but there are still reasons to embrace Gramsci’s oft-cited ‘optimism of the will,’ a 1929 dictum coined in a precursive period to the economic and political crises of our current time. As generations of health activists can attest, hopelessness has never been a good mobilizing strategy. The glass half-full will galvanize far more than the one half-empty, and 2015 presented the health promotion world with two such glasses: the COP21 Paris Agreement on Climate Change and the Sustainable Development Goals (SDGs). I will dispense with the Paris Agreement quickly. It is, unfortunately, replete with the permissive language of multilateral diplomacy, such as governments ‘aim to’ (rather than ‘will’), ‘should’ (rather than ‘shall’), ‘may’ (rather than ‘must’), and the ubiquitous sovereign escape clause: ‘in light of different national circumstances.’^8^ Such qualified ‘maybes’ renders the Agreement more supplicatory than compulsory, a glass less half-full than a leaky colander of questionable intents. But it is nonetheless a symbolic punctuation in global efforts to reduce or mitigate climate change, with many of the world’s governments substantially ramping up investments in alternative energy^9^ and the fossil fuel disinvestment movement accelerating. The Agreement’s half-full glass also tells us that climate change denialism is now relegated to dinosaur status, although whether this is too little too late remains a matter of huge and indeterminate import, as one might assume it was also for the reptilian giants of a past mass extinction.

## A Sustainable Agenda

By contrast, the SDGs are more hopeful, complex and potentially more forceful. Several years in the drafting with extensive public engagement, these post-Millennium Development Goals (MDGs) have been lauded as “a gigantic global version of Franklin Roosevelt’s New Deal, a plunge into public investment in order to stave off not just recession but also climate change, famine, and a few other horsemen of the apocalypse.”^10^ Others have been less generous in their assessment. The global economy’s voice of status quo, *The Economist*, described the SDGs as ‘stupid development goals’ that are ‘so sprawling and misconceived…that the entire enterprise is set up to fail.’^11^ One of the consistently cogent critics from the left, Patrick Bond, has labelled them ‘Seriously Distracting Gimmicks.’^12^ Both complaints have merit. Seventeen goals with 169 targets is certainly a sprawling agenda at a time when digitized attention spans seem to shrink with each new smartphone app. But the expansiveness of these goals are misconceived only if one holds to a market fundamentalism in which development (and a better life for all) is reduced to a simple matter of a few inexpensive interventions here and there. The SDGs, however, do risk becoming distractions if they remain abstracted from any analysis of how and why we are now living under ecological collapse and economic inequity^ [1] ^. In that respect, they are deeply, although not irrevocably, flawed.

Consider Goal 1, which commits governments to ending extreme poverty by 2030. *The Economist* likes this goal because it is simple and cheap; most of the progressive health and development community do not. The problem is the metric being used to measure extreme poverty ($1.25 PPP/day^ [2]^ now adjusted to $1.90 PPP/day), which is so low that it would leave those who achieve it living with huge shortages in the resources needed for a reasonable life expectancy. Based on this latter assumption (the ability to live in reasonable health to 70 years or so) the level of consumption needed rises to between $5.00 and $7.40 PPP/day.^14^ At the current rate by which the global economy has been reducing poverty it would take between 200 and 300 years to achieve this poverty reduction at these more meaningful levels.^15^ Even if this was eventually achieved, at what cost to all of the environmental SDGs if our prevailing economic growth model continues? The poverty example cuts to the contradiction at the heart of the SDGs: The implicit assumption that the same global economic rules that have created an increasingly unequal and unstainable world can somehow engineer the reverse.

## The World We Want

That is the glass half-empty, and there is no shortage of critique of the SDGs’ inconsistent targets and questionable indicators. But there remains a glass more than half-full. A total of 193 countries signed off on the SDGs; all the world’s leaders have given them the nod, however, much we might doubt their sincerity. Unlike the SDGs’ predecessor, the MDGs, *all* countries will have to account for progress on the new goals. The process of developing their ‘sprawling’ agenda involved extensive public input, captured in the 2013 United Nations (UN) Report, *A Million Voice: The World We Want*.^16^ The SDGs are, and should consistently be proclaimed as, the most comprehensive statement of how humans would like the world to be. The SDGs’ seeming utopian idealism that so bothers their critics is their very importance, since it portrays a world we would like to have, but do not and cannot have under a prevailing realpolitik that claims ‘politics’ as ‘the art of the possible.’ What we now need is a ‘politics of the improbable,’ one that focuses on some of the key goals and targets that, if heeded, would begin to undermine their contradictory and implicit embrace of an economic ‘business-as-usual.’

The SDGs are presumptively indivisible (all of them are to be pursued at the same time), which means that governments and multilaterals cannot cherry pick the ones they like and ignore the rest. Indeed, one of the targets for goal 17 specifically references the need for governments to ‘enhance policy coherence for sustainable development.’ The SDGs afford the strongest platform yet for governmental pursuits of ‘Health in All Policies,’ and chides somewhat the World Health Organization (WHO) for its dogged emphasis on Universal Health Coverage (just one of the health goal 3’s many targets) as its particular priority. The same argument of indivisibility exists for our several conventions on human rights, a more legalistic international agreement on how most of the world thinks we should be governed. Some human rights scholars, aware of the life-harming conditions facing many people, have applied moral reasoning (the ‘capabilities’ approach urged by authors such as Amartya Sen and Martha Nussbaum) to give priority to some rights over others.^17^ The same logic applies for the SDGs. Below are what I justify as the most immediately and enduringly important of the SDGs that could, and should, form the base for a post-Millennial health promotion platform, albeit not without correcting some of the critical weaknesses in each.

## Sustainable Development Goals and Health: The Short List

### Goal 1: End Poverty in All its Forms

Its importance to UN member states is signalled by its position as the first goal, and poverty is certainly the greatest single ‘risk condition’ for poor health. But the goal requires a meaningful metric and not the World Bank’s ‘extreme’ measure. The goal’s targets, however, usefully include implementation of social protection systems with ‘substantial coverage of the poor and vulnerable’ and ensuring that ‘all men and women, in particular the poor and the vulnerable, have equal rights to economic resources.’

### Goal 2: End Hunger, Achieve Food Security and Improved Nutrition and Promote Sustainable Agriculture

Undernourished people cannot be healthy or economically and politically well-functioning citizens. Apart from malnutrition targets, the goal calls for ‘sustainable food production systems …that increase productivity and production [and] that help maintain ecosystems.’ Emphasis is placed on assisting small-scale producers, but the targets are weak on eliminating derivatives (speculation) in food commodity markets.

### Goal 3: Ensure Healthy Lives and Promote Well-Being for All at All Ages

‘Achieve universal health coverage,’ including ‘affordable essential medicines and vaccines for all,’ is important, if weakened by disagreements over how it be financed (public or private or both?). ‘Universal access to sexual and reproductive healthcare services’ is equally important for its positive health impacts on women’s and children’s health, and its ability to keep population growth within ecological limits. But most of the targets concern reductions in mortality and morbidity rates with little discussion of how this might be achieved.

###  Goal 4: Ensure Inclusive and Equitable Quality Education and Promote Lifelong Learning Opportunities for All 

The link between education and good health are well-established, particularly for women and girls in low- and middle-income countries (LMICs).^18^ An important related target emphasizes development of knowledge and skills related to ‘sustainable lifestyles, human rights, gender equity…and global citizenship’ which, if well-implemented, could help to build a stronger activist base essential to moving governments forward on the SDGs. Without these targets, Goal 5’s aim to ‘Achieve gender equality and empower all women and girls’ is unlikely to succeed.

###  Goal 6: Ensure Availability and Sustainable Manage of Water and Sanitation for All

No water, no health; poor sanitation, much disease. This goal is the mainstay of historic public health, but its prioritization needs to be tempered with some critique. The first target references ‘affordable drinking water,’ code for engaging private markets in water supply or user fees for public provision. Recent history in both approaches has not been sanguine for equitable access.^19^ With diminishing supply and increasing population (and agricultural/industrial) demand, water access is becoming a source of conflict and a driver of refugee populations.

###  Goal 10: Reduce Inequality Within and Among Countries 

Given the impossibility of meaningful poverty reduction through economic growth alone (and where Goal 8’s promotion of ‘sustained…and sustainable economic growth’ ignores an almost 50 year history of environmental critique of the conventional growth model of the economy dating back to the Club of Rome’s 1972 *Limits to Growth,*^20^ the reducing inequality goal assumes paramount importance. It targets sustained income growth for the bottom 40% at a rate greater than the national average, but says nothing about the distortion of accumulating wealth at the top without which inequalities could continue to rise. Importantly it does add ‘equality of outcome’ to the usual emphasis on ‘equal opportunity,’ thereby affirming the more difficult of social justice’s two main articulations.^21^ The measure for ‘fiscal, wage and social protection policies’ that ‘progressively achieve greater equality’ is the labour share of gross domestic product (GDP) – of fundamental importance given the gross erosion in this share over the past 40 years of neoliberal economic policies.^4^ A disappointment was removal of a proposed global financial transaction tax, long opposed by the United States, the United Kingdom, and other countries with vested interests in footloose global capital.^4^

### Goal 12: Ensure Sustainable Consumption and Production Patterns 

The implication of this goal is more profound than its targets, which recycle most of the tropes of ‘sustainable development’ that first made the global rounds with the 1987 publication of the Bruntland Report, *Our Common Future*,^22^ adding ecology to the 1986 *Ottawa Charter’s* sociology.^23^ Weak on reducing fossil-fuel subsidies (another sovereign escape clause, ‘in accordance with national circumstances’), the goal itself needs a syntax inversion: not ‘sustainable consumption’ (in which ‘sustainable’ is a second place adjective to the primary nouns of consumption and production), but ‘consume sustainably’ (which can only be achieved by reducing current global levels of our material gorging). This recasting of the goal implies a reduction in demand, especially in HICs, at a time when conventional economics is calling for an increase in demand to get the growth economy back on track, underpinning again the foundational importance of an appropriately calibrated inequality goal.

## Sustainable Development Goals and Health: The Even Shorter List

This short list of SDG priority goals^ [3]^ does not mean that the ‘indivisible’ rest are ignored, but a campaign for all 17 goals and 169 targets runs the risk of petering away in a cacophony of special issues, in much the same way the ‘Occupy’ movement became diluted with scores of (albeit important and justifiable) claims that overwhelmed the initial focus on global economic financialization, predatory capitalism, government collusion/de-regulation, and the ignominy of a 1%. If health promotion 30 years post *Ottawa Charter* is still to embrace its ‘advocate’ role, its messaging on these basic ‘prerequisites to health’ (which is how I consider most of the SDGs) needs to be kept simple. To that end I would condense my own priority SDGs to just three.

1. Ensure quality education for women and girls (not to ignore men and boys, but emphasizing women and girls can rapidly advance gender empowerment, one of the best known means to improve health equity).
2. Reduce inequality (which in itself should eliminate poverty).
3. Consume and produce sustainably (which requires more equitable global patterns alongside aggregate global reductions, underpinning all the environmental SDGs).

But we need also to address *how* the SDGs might be achieved, the analytical piece missing from the final UN SDG document. If we assume that global capitalism will be around for a while longer, the only means that societies have so far found that can blunt its economic inequities and ecological damages have been:

- Increase the share of economic wealth going to labour (over capital).
- Increase progressive taxation, income redistribution and subsidization of public services and goods.
- Regulate the market for level playing field that is just and environmentally sustainable.

### Increasing Labour’s Bargaining Power

Although the first means is hinted at in one of the indicators for goal 10 on inequality (the labour share of GDP), the SDGs are silent on how this share might increase. Historical and contemporary evidence irrefutably posits the need for stronger (not weaker or more flexibilizing) labour and social protection legislation to prevent what Robert Reich calls the market’s ‘pre-distribution’ upwards to the rich,^25^ and which two International Monetary Fund (IMF) economists model conclude can only be effectively rectified by “restoration of the lower income group’s bargaining power.”^26^ Achieving this means a reversal of the current trajectory of informalization of the world’s labour force, which predominates in most LMICs and is experienced as ‘precarious employment’ in HICs (the on-call, no benefits, part-time work that has been increasing steadily in the past decade, and more rapidly post-2008 global financial crisis).^27,28^ More optimistically, the International Labour Organization (ILO) in 2015 adopted a new labour standard specifically targeting improvements for workers in the global informal economy.^29^

### Post-market Redistribution

The three sub-themes of the second imperative are not new. Post-market redistribution is the fastest and most efficient way to reduce or eliminate poverty.^30^ Such redistribution requires public investments in the goods and services essential for health and social cohesion, as well as direct income transfers. Income (cash) transfers alone can quickly be re-appropriated by private markets (increased prices for essential goods and services), a risk largely avoided when such goods and services are publicly provided or heavily subsidized. Both modes of redistribution rely upon deep public pockets which can only be filled (equitably) through progressive income and wealth taxation. The unprogressive extent to which taxes on wealthy individuals and corporations have declined in recent decades, along with overall global taxes as a percentage of world GDP, is staggering, and another reason for the rise of the 1%. A recent calculation using World Bank data estimates that the amount of untaxed income globally has more than doubled in the past decade, with over US$30 trillion more escaping taxation in 2012 than in 2002.^4^ As the latest leak on tax havens (the Panama Papers) shows, wealthy individuals and transnational companies have (immorally and also frequently illegally) escaped taxation while relying upon publicly provided infrastructures to generate their wealth. So far governments have been reluctant to renounce global tax competition (keep rates low to avoid capital flight to lower regime countries), close the egregious offshore financial centres, or even (despite the nominal support of some 65 countries) enact a global financial transaction tax (the one initially proposed for goal 17). If such a tax were levied on all currency transactions at a virtually unnoticeable rate (except for those whose wealth is generated by volatile, speculative investing) it would generate over US$8 trillion annually, more than enough to fund the estimated US$4.5 trillion needed annually to finance the SDGs.^31^ Re-appropriated public revenues through progressive national and new global forms of taxation could enrich existing global funding mechanisms supporting some of the SDGs, or new ones created to finance the others.

### Regulating for a Level Playing Field

The final imperative is an essential tonic to the ‘green washing’ of corporate social responsibility (CSR), in which the pursuit of voluntary self-regulation of risks to environmental, social or human health is advanced over the ‘second-best’ option of government (or multilateral and enforceable) regulation. Alongside goal 17’s uncritical embrace of public-private partnerships (PPPs) and the WHO’s 2016 controversial adoption of a Framework for Engagement with Non-State Actors (FENSA), critiqued as opening the doors to corporate lobbying, financing and influence, the allure of ‘multi-stakeholder governance’ rests on the same premise of the SDGs’ core contradiction: That the forces of wealth, power and privilege will munificently act for the betterment of all. It is beyond the scope of this commentary to plumb the contentious depths of PPPs^32^ and their CSR cousin. Suffice it to say that the interests of capital accumulation, even when pursued for ‘social impact investing’ (the Rockefeller Foundation’s latest big idea) where venture capitalists invest in a social good for profitable return,^33^ will continue to exacerbate the very wealth inequalities that underpin our present economic and ecological crises. Invoking even conventional economics, relying upon voluntary self-regulation runs the risk of penalizing corporations that attempt to do a little good while its less responsible competitors ‘free-ride’ to gain market advantage. The concept of a level playing field requires rules of competition that are the same for all market players, which cannot be achieved without enforced regulations for the public good.

## Too Grand a Health Promotion Agenda?

For health promoters more comfortable with the *Ottawa Charter’s* ‘enable and mediate’ strategies, the prospect of thinking and acting (advocating) globally may seem a bit daunting. There are three important reasons why it need not be.

First, most of the policy changes required to make the world healthier and survivable start at the national level. It is national governments that created the global economy in its present shape, and which can restrain (at least) part of its future toxicity. It is national governments that are responsible for implementing the normative grandeur of the SDGs, and to ensure that their domestic and foreign policies support their attainment. Although some of the SDG targets and indicators are weak in terms of where we need to go to ensure health equity, they are a base from which to demand more.

Second, there is no shortage of evidence that supports the shorter list of key SDGs and the three advocacy imperatives. Evidence alone does not speak truth to power, but it does lend power to truth-speakers.

Third, our engagement in such advocacy (politely through the channels of participatory democracy, more stridently through social media and campaigns, civilly disruptive in protest) is something to be undertaken not simply as health promotion researchers or practitioners; it is a right and responsibility of our citizenship. This may strike some as a simplistic claim and, glass half-empty, there is little reason to expect that such actions will guarantee the results we seek, ie, governments keen to implement the SDGs, to strengthen labour over capital, to redistribute resources progressively and to regulate for a fairer future. But the glass half-full tells us that not doing so simply guarantees our failure.

There will be distractions in our health promotion efforts along the way. One is the long-standing risk of ‘lifestyle drift’ in which individual or ‘target group’ behaviour change is given preference over efforts to shift the political economy into one that creates healthier living conditions and, as older health promotion parlance puts it, ‘makes healthy choices the easy choices.’^34^

A second distraction is development’s newfound infatuation with ‘resilience,’ a term that appears 14 times in the SDGs, has over 47 million Google entries, and has seen Web of Science citations increase from almost zero in 1997 to nearly 30 000 in 2015.^35^ Resilience is seen as a positive re-framing of ‘vulnerability’ in its emphasis on ‘making people, communities and systems better prepared to withstand catastrophic events (both natural and manmade) and able to bounce back more quickly and emerge stronger from these shocks and stresses.’^36^ This is reminiscent of health promotion’s infatuation in the 1990s with ‘community capacity mapping,’^37^ in which community health workers started locating deprived communities’ assets and self-organizing strengths. Resilience and capacity mapping share in common a glass-near-full view of the world, potentially valuable in galvanizing the hopefulness of change; but both risk losing sight of, and actions on, the structural determinants of oppression, injustice and environmental collapse. SDG 1’s target committing governments to ‘build the resilience of the poor’ in relation to ‘economic, social and environmental shocks and disasters’ would have more salience if the suite of SDGs were stronger in identifying the sources of, and solutions to, those same shocks and disasters.

A final distraction resides in another recent obsession: innovation, which makes nine separate appearances in the SDGs. Generally applied to the need for a post-industrial ‘innovation economy,’ when invoked for health innovation could represent the pursuit of novelty without appreciating that tried and true approaches might still have worth; or that innovative health technologies should first ask why older ones have not been successful, since often the problem is with the politics of their use, and not their utility.

## Another Health Promotion Declaration

In May 2016, the WHO released its ‘zero draft’ *Shanghai Declaration on Health Promotion in the 2030 Agenda for Sustainable Development*^38^ to be finalized and released following the ninth global health promotion conference to be held in Shanghai in late November, 2016. Declarations are amongst the weakest forms of international norm-setting (with no enforcement or even reporting obligations) but can offer support to activists within and outside of governments in legitimating novel interventions or much needed advocacy. In that respect, the zero draft captures many of the critical points related to the SDGs raised above. It recognizes that ‘inequalities between and within countries, in income levels, opportunities, and health outcomes, are now greater than at any time in recent decades,’ and that, combined with an ‘increase in violence, the force of unsustainable production and consumption, and the negative impacts of climate threats…stand in the way of a better life and health for all.’ It obliquely critiques the current ‘uneven socio-economic development’ model for fomenting the rise in ‘non-communicable diseases, mental health and environmental diseases,’ and references the negative impacts of globalization of marketing and trade in ‘tobacco and alcohol, and food products and sugary drinks not consistent with a healthy diet.’ Social movements are claimed to ‘have gained momentum in advocating change’ while ‘social mobilization’ is applauded for leading ‘to the demand by citizens for better health…and provid[ing] them with a meaningful voice.’ Governments are challenged to ‘expand the space for all people to participate through community-centred approaches…and also politically,’ and reiterates health promotion’s long-standing intent to ‘empower citizens,’ albeit now recognizing the need for ‘strong involvement of civil society’ in any such process.

In these respects the zero draft is punchier in tone than the more anodyne SDGs. But while recognizing the need to ‘protect public health from undue influence by any form of vested interest’ the draft ignores how new trade and investment agreements are doing the opposite for heavily vested corporate interests.^39^ Its embrace of social movement activism and call to increase citizens’ political voices grates against the actions of an increasing number of states actively suppressing such aims.^40^ Unintentionally ironic, the zero draft further credits the ‘guidance’ of the People’s Republic of China with five major development ‘notions,’ including ‘openness and sharing.’ While China’s Gini coefficient for income distribution has dropped slightly from a high of 0.49 in 2012 to 0.46 in 2015^41^ (anything over 0.40 is considered by the UN to be a recipe for social unrest) it is a far cry from its 1980’s pre-market reforms score of 0.3. The country now has more dollar billionaires than the United States.^42^ With respect to openness, Reporters without Borders in 2015 ranked China 176 out of 180 on its index of press freedom,^43^ a drop in one place since 2014. Beautifully crafted notions are no substitute for substantive action.

Therein lies the Declaration’s weakness. It laments many of the world’s most serious and health-compromising crises but pulls back in a muted call to ‘strengthen good governance for health,’ ‘improve urban health,’ and ‘strengthen health literacy.’ For health promoters aware of the urgent need for a more assertive politics of hope (and change), the zero draft offers little apart from its emphasis on curbing trade and investment in unhealthy commodities, ensuring fiscal space for strong public health systems, and calling for a ‘Health in All Policies’ approach to government actions across the SDGs. Good as far as these commitments go, a rallying cry for a new environmentally sustainable and economically just political order they are not.

## Conclusion

The occasion of the *Ottawa Charter’s* 30th anniversary has created a personal déjà vu moment. Five years ago in a Supplement in the journal, *Health Promotion International*, I celebrated the *Ottawa Charter’s* 25th anniversary by urging an advocacy platform remarkably similar to the one elaborated upon above.^44^ I exhorted health promoters to support social movements and civil society activists working at national and global scales to pressure for economic reform and ecological salvation. I called for a re-valorization of the social state and progressive taxation as antidote to apathy and global corporatization. I cautioned strongly on the need to combat xenophobia, the misplaced antipathy with a neoliberal globalization that has failed most people. The ultra-nationalist and anti-migrant sentiment, deliberately stoked by some pugnacious political opportunists, is more deeply troubling today than it was just 5 years ago, as the number of our planetary brethren displaced by war and persecution and seeking safe refuge in another country rose to 65 million in 2015^45^ – to say little of those simply fleeing economic poverty and habitats made unlivable by climate change and population pressure.

Neoliberalism, the economic model that has predominated and slowly globalized over the past 40 years, may be on the way out, with voices from within its former bastions of promulgation (such as the IMF) now explicitly critiquing its excesses and failures.^46^ But it is useful to locate neoliberalism’s rise (and perchance fall) within the episodic crises of capitalism itself. When the neoliberal model of free trade, market fundamentalism, minimal government interference, and the de-regulated free movement of capital first gained a political foothold in the late 1970s and early 1980s, it was partly in response to another round of capitalist economic recessions and stagflation in HICs and the foreign debt crisis in LMICs.^4^ Neoliberalism’s economic proponents may have deeply believed in the theoretical correctness of their policy nostrums, but economic elites and conservative politicians also saw how these nostrums could enrich their own wealth and power after the equalizing three decades that followed the Second World War. We are left, then, with an older political dilemma, drawn more sharply since the 2008 financial crisis but deepening with each passing year: How can we tame capitalism and its predatory market logic to support human equity and (now) a livable planet? Or, if it cannot be tamed (its resilience – and this is a good instance in which to invoke that term – is remarkable), how might capitalism be transformed into something better fit for human social and ecological survival into a 22nd century?

And so I closed my 25th anniversary article with thoughts still pertinent today:

The deeply structural forms of health-promoting change we so urgently need are only likely to arise in the wake of even more profound crises. Our task, as we continue our quotidian and localized best health promoting efforts, all the time supporting those attempting to leverage change at national and global levels, is to nurture the blueprint for what a social order could look like, if human, animal and ecological health formed its core rather than being relegated to its periphery.^44^

This task is now slightly easier, with the SDGs providing such a blueprint upon which we can (and must) build.

## Ethical issues

Not applicable.

## Competing interests

Author declares that he has no competing interests.

## Author’s contribution

RL is the single author of the paper.

## Endnotes


[1] In describing our present time as one of economic inequity, I am not ignoring that the rise of a middle class in China (in particular), India and, to a lesser extent, other developing nations has led to some reduction in global income (but not wealth) inequalities between individuals since 2000, albeit with inequalities remaining extraordinarily immense. More importantly, income inequality *within* most nations continues to rise, and it is at this national scale that its threats to social cohesion, health and development are most serious.^13^

[2] PPP (purchase power parity) is an adjustment made to estimate the cost in US$ of a selected basket of goods in all the world’s countries. It is promoted primarily by the World Bank and, though not without criticisms, remains widely used as an estimate of poverty.

[3] My priority list differs somewhat from a list of health-related SDGs developed by the WHO, which are more narrowly focused and less politically critical.^24^

